# Temporal Orienting of Attention Can Be Preserved in Normal Aging

**DOI:** 10.1037/pag0000105

**Published:** 2016-06-13

**Authors:** Joshua J. Chauvin, Celine R. Gillebert, Gustavo Rohenkohl, Glyn W. Humphreys, Anna C. Nobre

**Affiliations:** 1 Department of Experimental Psychology, Brain and Cognition Laboratory, University of Oxford

**Keywords:** aging, temporal orienting, expectation, visual attention, top-down control

## Abstract

Being able to orient our attention to moments in time is crucial for optimizing behavioral performance. In young adults, flexible cue-based temporal expectations have been shown to modulate perceptual functions and enhance behavioral performance. Recent studies with older individuals have reported significant deficits in cued temporal orienting. To investigate the extent of these deficits, the authors conducted 3 studies in healthy old and young adults. For each study, participants completed 2 tasks: a reaction time (RT) task that emphasized speeded responding and a nonspeeded rapid-serial-visual-presentation task that emphasized visual discrimination. Auditory cues indicated the likelihood of a target item occurring after a short or long temporal interval (foreperiod; 75% validity). In the first study, cues indicating a short or a long foreperiod were manipulated across blocks. The second study was designed to replicate and extend the first study by manipulating the predictive temporal cues on a trial-by-trial basis. The third study extended the findings by including neutral cues so that it was possible to separate cueing validity benefits and invalidity costs. In all 3 studies, cued temporal expectation conferred significant performance advantages for target stimuli occurring after the short foreperiod for both old and young participants. Contrary to previous findings, these results suggest that the ability to allocate attention to moments in time can be preserved in healthy aging. Further research is needed to ascertain whether similar neural networks are used to orient attention in time as we age, and/or whether compensatory mechanisms are at work in older individuals.

Our brains continuously generate expectations about what we are about to see, touch, taste, or hear. These predictions operate to guide and enhance our behavioral performance, which in turn enables us to interact effectively with our complex and ever-changing environment. At the core of our predictive capabilities is our ability to orient our attention proactively to key moments in time—enabling us to ready ourselves to perceive and respond to relevant events. Our abilities to orient ourselves in time and form expectations about the world can lead to improved behavioral outcomes, such as faster response times, greater accuracy, and increased perceptual sensitivity in perceptually demanding conditions (for a review, see Nobre & Rohenkohl, 2014).

Predictive temporal cues provide an effective means of manipulating temporal expectations that are under top-down control (Nobre & Coull, 2010; Rohenkohl, Coull, & Nobre, 2011). Just as spatial cues can be used to direct a participant’s attention to specific locations (Posner, 1980), temporally predictive cues have been used to manipulate participants’ expectations and guide their attention to key moments in time when a task-relevant target is likely occur (Correa, Sanabria, Spence, Tudela, & Lupiáñez, 2006; Coull & Nobre, 1998; Miniussi, Wilding, Coull, & Nobre, 1999; Todorovic, Schoffelen, van Ede, Maris, & de Lange, 2015; Triviño, Correa, Arnedo, & Lupiáñez, 2010). The use of temporal cues to direct an observer to a moment in time has been referred to as *temporal orienting* (see Nobre, 2001, for review). Temporal orienting appears to be a flexible ability that is not only capable of speeding motor preparation and response times (Correa, Lupiáñez, Milliken, & Tudela, 2004; Griffin, Miniussi, & Nobre, 2001), but can also improve the perceptual sensitivity to detect or discriminate stimuli (Correa, Lupiáñez, & Tudela, 2006; Correa, Lupiáñez, & Tudela, 2006; Davranche, Nazarian, Vidal, & Coull, 2011; Rohenkohl, Cravo, Wyart, & Nobre, 2012).

Whereas in younger adults cued temporal orienting has consistently been shown to optimize behavioral performance in speeded motor tasks as well as in unspeeded, perceptually demanding tasks (Correa et al., 2004; Coull & Nobre, 1998; Davranche et al., 2011; Miniussi et al., 1999), the extent to which temporal orienting is preserved in healthy aging is a matter of debate (Zanto & Gazzaley, 2014), Significant deficits have indeed been reported for older adults in cued temporal orienting tasks (Dempster, 1992; Zanto et al., 2011): Zanto and colleagues (2011) directly manipulated temporal expectation in young and old adults across a range of tasks. Although younger adults were able to use temporal cues to enhance RTs in a detection task, a forced-choice discrimination task and a go/no-go discrimination task, older adults did not gain any benefit from temporal cuing in any of the task conditions. The researchers suggested that older adults have a deficit in temporal expectation, as evidenced by their inability to use temporal cues to successfully allocate attentional processes in time. Based on such results it has been argued that deficits in temporal orienting exist as part of a wider set of problems related to reduced, proactive top-down control of attention (Zanto & Gazzaley, 2014). In the following set of experiments, we investigated the generality of these deficits, by asking again whether, in some cases, temporal orientation can be preserved in healthy aging.

To gain a clearer picture of the boundaries of temporal expectation abilities and deficits, younger and older adults participated in two types of tasks, emphasizing speeded responding and perceptual discrimination. The task designs were adapted from those used by Davranche et al. (2011; see also Correa, Lupiáñez, & Tudela, 2006). A speeded RT task emphasized the effects of orienting on response preparation. This task demanded a speeded response, and did not require participants to discriminate between detailed features of a target item. Separately, a rapid serial visual presentation (RSVP) task emphasizing perceptual discrimination was used to examine the temporal expectation effects associated with perceptual processing. Participants made a nonspeeded response to indicate whether an X or O target appeared in the preceding stream of letters. Temporal expectations were manipulated by auditory cues that predicted the onset time of target stimuli with 75% validity. Participants were instructed to make use of the audio cue (either high pitch, or low pitch) to help them attend to a point in time.

Based on the sparse previous literature (for review, see Zanto & Gazzaley, 2014), we hypothesized that older adults would show significant deficits in using temporal orienting cues. Our study was designed to extend the previous literature by examining whether temporal orienting effects could be unmasked in older adults by reducing executive demands in the task. Complex or demanding tasks could, in theory, compromise performance in the elderly for reasons unrelated to deficits in deriving or using temporal expectations. In addition, by including the two types of experimental paradigms, we were able to examine whether modulation of motor or perceptual functions by cued temporal expectations could be differentially preserved in healthy aging.

Three experiments were conducted using variations of these two tasks. In the first experiment, we presented blocks of trials where the audio cues predicted either a short (540 ms) or a long (1,600 ms) interval until the target appeared. We chose to block the cues to isolate putative effects of temporal orienting, because blocking of cues reduces the demands on other executive processes that are not specifically linked to temporal orienting, such as interpretation and updating of the cue information. In this task, the group of older participants showed significant benefits of temporal orienting cues for both the speeded RT and RSVP tasks. In a second experiment, we increased task demands by intermixing cues predicting short and long intervals. This allowed us to examine whether older adults could still benefit from temporal orienting cues when they had to rely on executive functions related to encoding and updating the meanings of cues on a flexible basis. Temporal orienting deficits in this case would suggest that these additional executive demands might have contributed to the temporal expectation deficits observed in previous studies. However, the older participants in this study still showed significant benefits of temporal orienting cues for both the speeded RT and RSVP tasks. In the third and final experiment, we included blocks of noninformative temporal cues in addition to blocks of temporally predictive cues in order to separate validity benefits from invalidity costs. As in the two previous studies, the older group showed significant effects of cued temporal orienting, which consisted of both validity benefits and invalidity costs.

## General Methodology

### Participants

Each experiment consisted of 18–20 participants in both younger and older groups. Each participant took part in only one experiment, with the exception of 13 older participants who took part in Experiment 1 and Experiment 2. All experiments were conducted more than 6 months apart. All participants self-reported to be right-handed, with the exception of four older (one in Experiment 1, three in Experiment 3) and two younger (in Experiment 3) participants, who were left-handed. The participants all reported normal or corrected-to-normal vision, were free of psychotropic or vasoactive medication, and had no neurological or psychiatric history. Volunteers gave informed consent and were reimbursed for their participation (£10 an hour plus travel expenses). The studies were reviewed and approved by the Central University Research Ethics Committee of the University of Oxford.

### Neuropsychological Testing

To ensure that our older participants were cognitively healthy, we evaluated their performance using a neuropsychological test battery. In Experiment 3, we performed the neuropsychological test battery for both older and younger participants. The neuropsychological evaluation consisted of tests designed to assess general cognitive function (Montreal Cognitive Assessment [MoCA], version 7.1; Nasreddine et al., 2005), attention/task switching (Trail Making Test [TMT]; Reitan, 1958; Reitan & Wolfson, 1985; Tombaugh, 2004), executive function (Rey-Osterrieth Complex Figure Test [ROCFT]; Fastenau, Denburg, & Hufford, 1999; Meyers & Meyers, 1995; Osterrieth, 1944; Rey, 1941), semantic memory (category fluency—names of animals only; Gladsjo et al., 1999), verbal language/verbal memory (Hopkins Verbal Learning Test [HVLT]; Brandt, 1991; Vanderploeg et al., 2000), language/semantic memory (15 Boston Naming Test [BNT]; Mack, Freed, Williams, & Henderson, 1992), verbal working memory (Digit span; Wechsler, 2008), premorbid IQ (Test of Premorbid Functioning [TOPF]; Wechsler, 2009), and motor function (Purdue Pegboard; Desrosiers, Hebert, Bravo, & Dutil, 1995; Yeudall, Fromm, Reddon, & Stefanyk, 1986). The test scores are summarized in Tables 1 and 2.[Table-anchor tbl1][Table-anchor tbl2]

### Apparatus

Stimuli were created and presented through Presentation (16.5, Neurobehavioural systems, Albany, CA, United States of America), run on a Dell Optiplex 990 computer with a 23-inch ViewSonic VA2342-LED screen (resolution 1920 × 1080 pixels, refresh rate 100 Hz). Participants were seated in a dimly lit room, approximately 63 cm away from the monitor. Responses were collected using a standard keyboard.

### Stimuli and Tasks

**Figure 1 fig1:**
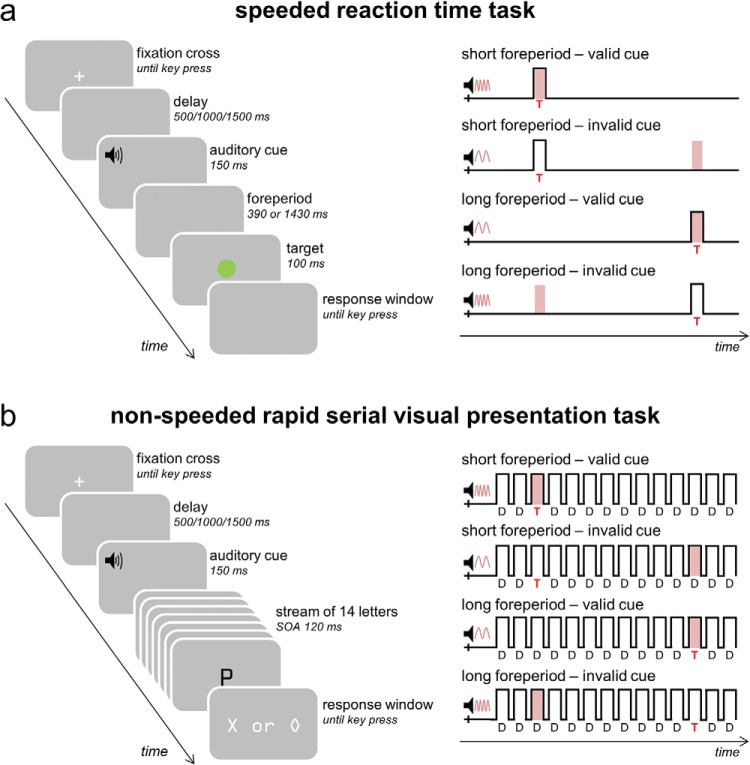
Schematic illustration of the speeded reaction time (RT) task and the rapid serial visual presentation (RSVP) task. Auditory cues predicted when target events were more likely to occur. (a) Speeded RT task. Targets consisted of green circular patches presented foveally. Participants were instructed to respond as quickly as possible to the green patch by pressing the left arrow key on a standard keyboard with their right index finger. (b) RSVP task. Targets were either an X or an O that was presented foveally. Participants were instructed to hold off on responding until the end of the trial, and to press the left arrow key if they thought they saw an X and the right arrow key if they thought they saw an O. See the online article for the color version of this figure.

Each experiment consisted of a speeded RT task and an RSVP task. In the speeded RT task (Figure 1a), participants were instructed to respond as quickly as they could to a green circular patch, which appeared at the center of the screen. In the nonspeeded RSVP task (Figure 1b), participants were instructed to identify a target letter (X or O) embedded in a stream of distractor letters. In both tasks, the pitch of an auditory cue preceding the target indicated the likelihood of the target item occurring after a short (540 ms) or long (1,580 ms in the speeded RT task and 1,620 ms in the RSVP task) temporal interval (75% validity). Participants were instructed to maintain central fixation throughout the tasks and to do their best to use the temporal information provided to them by the auditory cues to help them to predict when the target was most likely to appear.[Fig-anchor fig1]

Both tasks followed the same basic design (see Figure 1). Stimuli appeared superimposed against a uniform gray background (RGB values: 128, 128, 128), and a fixation point remained visible in the center of the screen (width: 0.46°; height: 0.46°). Each trial commenced following a participant-initiated key press. After a short delay lasting 500 ms (50% probability), 1000 ms (25% probability), or 1,500 ms (25% probability), an audio cue was presented for 150 ms. In Experiments 1 and 2, the audio cue was either a high pitch (1,100 Hz) or a low pitch (600 Hz) beep indicating a short or a long foreperiod, respectively. The cue was valid in 75% of the trials. Participants were informed that the audio cues would help them to predict when the target would appear. In Experiment 3, we again used a high pitch (1,600 Hz) and a low pitch (400 Hz) beep indicating a short or a long foreperiod with 75% validity. In addition, we introduced an audio cue with an intermediate pitch (1,000 Hz) as a neutral cue that provided no information about the duration of the foreperiod.

In the speeded RT task, participants were asked to respond as quickly as possible with their right index finger to a green circular patch (diameter: 1.82°), which was presented foveally after either a short (stimulus onset asynchrony [SOA] of 540 ms) or a long foreperiod (SOA 1580 ms; Figure 1a). In the nonspeeded RSVP task, the audio cue was followed by a stream of 14 black letters (font: OCR A Extended; width: 0.9°; height: 1.92°) presented foveally and in rapid succession (duration 100 ms; interstimulus interval 20 ms). The SOA between the audio cue and the first letter was 300 ms. Thirteen letters were distractors and one was a target letter (Figure 1b). The target letter, either an X or an O, appeared either early (on the third location, after 540 ms) or late (on the 12th location, after 1,620 ms). The distracter stimuli were randomly sampled without replacement from a set of letters (A,B,E,F,G,H,I,J,L,M,P,Q,R,T,U,W). Following the presentation of the letter stream, participants made a nonspeeded, delayed discrimination response with their right hand using the left (for X) and right (for O) arrow keys on a standard keyboard. To minimize the motor component of the perceptual discrimination task, participants responded after the offset of the visual stream during a designated response window. Participants were under no time pressure to provide a response and were informed that only the accuracy of the response would be taken into account.

### Data Analysis

Statistical analysis was performed using MATLAB and SPSS. For the speeded RT task, our primary outcome variable was the mean RT on correct responses for each condition. Trials were excluded from the analysis if the RT was more than three standard deviations above the mean RT. The average number of outlying trials was low (1%) and did not differ between young and old adults (see Table 3). To ensure that age-related effects were not related to general slowing of older compared to younger adults, we also calculated for each foreperiod a cueing index. For Experiments 1 and 2, the index was calculated by taking the difference between the mean RT in the invalid condition and the mean RT in the valid condition, and dividing this difference by the mean RT in the valid condition. For Experiment 3, we calculated one index reflecting validity benefits (the mean RT in the valid condition minus the mean RT in the neutral condition, divided by the mean RT in the neutral condition), and one index reflecting invalidity costs (the mean RT in the invalid condition minus the mean RT in the neutral condition, divided by the mean RT in the neutral condition). In a supplementary analysis, we analyzed the proportion of anticipatory responses (see Supplementary Materials).[Table-anchor tbl3]

For the nonspeeded RSVP task, our primary outcome variable was a measure of perceptual sensitivity (*d*′). Trials were excluded from the analysis if the RT was more than three standard deviations above the mean RT (see Table 3). In addition, although response speed was de-emphasized, we also analyzed the mean RTs on correct responses for each condition for the sake of completeness (see Supplementary Materials).

For each measure, we excluded from the analysis data from participants who scored more than three standard deviations away from the mean value in at least one condition.

To examine how sensitivity to temporal prediction changed with age, we ran a 3-way mixed-design analysis of variance (ANOVA) with foreperiod (short, long) and cue validity (Experiments 1 and 2: valid, invalid; Experiment 3: valid, invalid, neutral) as within-subjects factors, and age group (young, old) as a between-subjects factor for each task. When sphericity could not be assumed (Mauchly’s sphericity test: *p* < .05), *p* values were adjusted using the Greenhouse-Geisser correction (G-G correction).

As part of a supplementary set of analyses, to assess whether differences in performance depended on whether the auditory cues were blocked, we ran a four-way analysis of variance with experimental design (blocked design, trial-by-trial design) as between-subjects factor. Only older participants who participated in Experiment 1 and in Experiment 2 were included in the analysis (*n* = 13; see Supplementary Materials).

Finally, to account for the possibility of unequal trial numbers or power between the trial conditions or groups, we conducted a series of nonparametric permutation tests to analyze the strength of the validity effects for each experiment (Ernst, 2004; Maris, Schoffelen, & Fries, 2007; see also Rohenkohl, Gould, Pessoa, & Nobre, 2014). To this end, we performed repeated-measures ANOVAs separately for young and old adults. Statistical tests used a critical alpha level of 0.05. We assessed the significance of the observed results by comparison to a null distribution generated via Monte Carlo simulation with 10,000 repetitions. This null distribution was created by randomly shuffling the condition labels within each participant’s data in each repetition. We then performed the statistical test (*F*), and the resulting value was entered into the null distribution. The permutation *p* value was determined as the proportion of random partitions that resulted in a larger test statistic than the observed one.

## Experiment 1: Temporal Orienting in a Blocked Design

### Method

Eighteen younger participants (*M*_age_ = 26.8 years, *M*_education_ = 21.5 years, 10 females) and 18 older participants (*M*_age_ 65.5 years, *M*_education_ = 18.0 years, 10 females) took part in the experiment. In this experiment, the pitch of the audio cue was manipulated between blocks. In half of the blocks, a high-pitched auditory cue was presented on every trial indicating that the target would appear after a short interval with a probability of 75%. In the other blocks, a low-pitched auditory cue occurred indicating that the target would appear after a long interval with a probability of 75%. Participants performed four blocks consisting of 96 trials each for each task. Two blocks of the speeded RT task were alternated with two blocks of the RSVP task. A short practice block was given before each set of two blocks. The order of the tasks was counterbalanced across participants.

### Results and Discussion

#### Speeded RT task

After removing the anticipatory responses, performance was at ceiling (<1% misses) for all four conditions in both age groups. Before analyzing the between-groups differences, two participants (one young, one old) with response times more than 3 *SD*s above the average response time of all the other participants were removed from the analysis.

**Figure 2 fig2:**
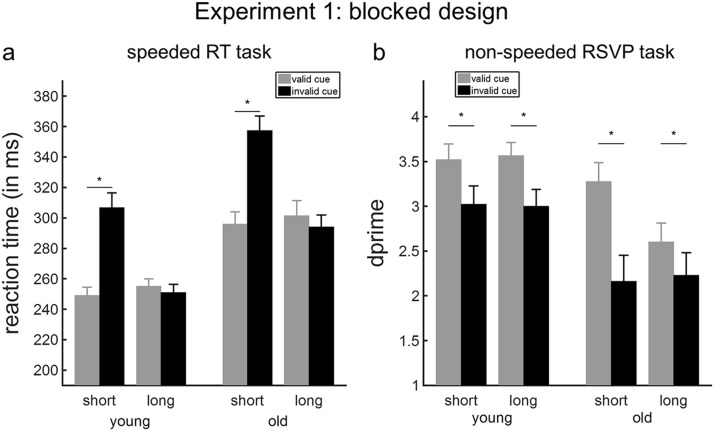
Temporal orienting effects in Experiment 1 (blocked design). (a) Effects of temporal expectations on reaction time (RT) values (ms) in the speeded RT task. (b) Effects of temporal expectations on sensitivity scores (*d*′) to the target items in the rapid serial visual presentation (RSVP) task. Error bars represent SEM. Asterisk values denote significant effects.

The key results of the ANOVA are listed in Table 4. The main finding was that both younger and older adults showed significant and equivalent effects of cued temporal expectations on speeded detection of targets appearing at the short interval. We observed main effects of age, foreperiod, and cue validity; as well as a foreperiod-by-validity interaction on the RTs (*p*s < .001). Older participants responded more slowly to the target compared to younger individuals (Figure 2a). Post hoc paired-sample *t* tests were conducted to inform the foreperiod-by-validity interaction, which was significant within each age group: young adults, *F*(1, 16) = 54.42, *p* < .001, *F* test permutation *p* < .001; and old adults, *F*(1, 16) = 59.84, *p* < .001, *F* test permutation *p* < .001. Participants reacted significantly more quickly to targets appearing after a short foreperiod when the preceding cue contained valid versus invalid temporal information, *t*(33) = −11.92, *p* < .001. The effect size was very large in both age groups (young adults: Cohen’s *d* = 1.78; old adults: Cohen’s *d* = 2.32; Figure 2a). In contrast, the validity of the auditory cue did not modulate RTs in trials with a long foreperiod, *t*(33) = 1.76, *p* = .09. To ensure that the absence of any age-related differences in the validity effect was due to old adults responding more slowly than young adults, we additionally analyzed the ‘cueing index’ that was corrected for the mean RT of each individual. We did not observe a main effect of age, *F*(1, 32) = .22, *p* = .64 or an interaction between foreperiod and age, *F*(1, 32) = .18, *p* = .67. In summary, both younger and older participants experienced an asymmetric cueing benefit for short versus long foreperiods.[Table-anchor tbl4][Fig-anchor fig2]

#### RSVP task

No participant performed more than 3 *SD*s beyond the mean for any condition on perceptual discrimination, so all participants were included in the analysis. Analysis of *d*′ revealed significant and equivalent effects of blocked cued temporal expectations for detecting targets at the short and long intervals in both younger and older adults. We observed main effects of age and cue validity on the *d*′ values (*p*s ≤ .006, see Table 5). The main effect of cue validity was significant in each age group (young adults: *F*(1, 17) = 19.94, *p* < .001, *F* test permutation *p* < .001; old adults: *F*(1, 17) = 23.61, *p* < .001, *F* test permutation *p* < .001). As shown in Figure 2b, the target letter was identified better when the cue correctly predicted its position in the RSVP stream compared to when the cue was invalid. Although in general the performance of older participants was worse than that of younger participants, the size of the cue validity effect was large in both age groups (young adults: Cohen’s *d* = 1.05; old adults: Cohen’s *d* = 1.15).[Table-anchor tbl5]

In summary, in contrast to previous reports (e.g., Zanto et al., 2011), the results of Experiment 1 provide the first behavioral demonstration that cued temporal orienting can be preserved in healthy aging. Importantly, our experimental design differed from Zanto and colleagues, who manipulated temporal cues on a trial-by-trial basis. An event-related design may be more sensitive to pick up subtle deficits in the flexibility of cued temporal orienting, as participants need to change their temporal expectation on a trial-by-trial basis. In a second experiment, we therefore intermixed cues predicting short and long intervals. This allowed us to examine the extent to which executive functions related to encoding and updating the meaning of the cue might have contributed to the temporal expectation deficits observed in previous studies.

## Experiment 2: Temporal Orienting in a Trial-by-Trial Design

### Method

Eighteen young (*M*_age_ 25.0, *M*_education_ = 18.0 years, 11 females) and 20 older (*M*_age_ 66.8, *M*_education_ = 17.85 years, 12 females) volunteers took part in the experiment. In this experiment, high- and low-pitched auditory cues were randomly intermixed within each block. Participants completed for each task four blocks of 96 trials each. Two blocks of the speeded RT task were alternated with two blocks of the RSVP task. A short practice block was given before each set of two blocks. The order of the tasks was counterbalanced across participants.

The use of trial-by-trial cueing also permitted the analysis of sequential effects. Sequential effects refer to how the order of the foreperiod intervals can influence performance (Los, 2010). Previous studies found asymmetrical sequential effects, showing that RTs are lengthened when the previous foreperiod was longer than the current foreperiod (e.g., Capizzi, Correa, & Sanabria, 2013; Drazin, 1961; Los & Van Den Heuvel, 2001; Steinborn, Rolke, Bratzke, & Ulrich, 2008; Vallesi & Shallice, 2007). Performance on valid trials was analyzed using a 3-way mixed-design ANOVA with current foreperiod (short, long) and previous foreperiod (short, long) as within-subjects factors, and age group (young, old) as a between-subjects factor. Only validly cued trials preceded by a validly cued trial were included in this analysis.

### Results and Discussion

#### Speeded RT task

**Figure 3 fig3:**
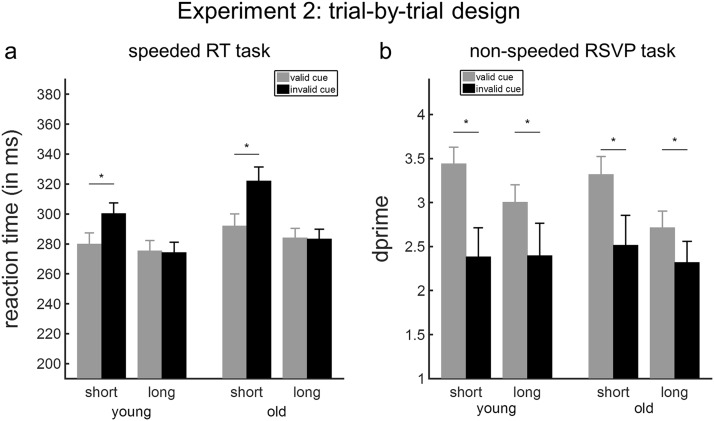
Temporal orienting effects in Experiment 2 (trial-by-trial design). (a) Effects of temporal expectations on reaction time (RT) values (ms) in the speeded RT task. (b) Effects of temporal expectations on sensitivity scores (*d*′) to the target items in the rapid serial visual presentation (RSVP) task. Error bars represent SEM. Asterisk values denote significant effects.

No participants were excluded from the RT analysis. Extending the results of the blocked-design version of the task in Experiment 1, significant and equivalent effects on the speed of detecting targets occurring at the short interval were also conferred by fully intermixed temporally predictive cues in both younger and older adults. Analysis of RTs revealed main effects of foreperiod and cue validity, and a significant interaction between foreperiod and cue validity (*p*s < .001, Table 4). We did not observe any significant difference between the age groups (see Table 4). Post hoc paired-sample *t* tests were conducted to inform the foreperiod-by-validity interaction, which was significant in young adults, *F*(1, 17) = 22.53, *p* < .001, *F* test permutation *p* < .001, and in old adults, *F*(1, 19) = 23.64, *p* < .001, *F* test permutation *p* < .001. Responses to targets appearing after a short foreperiod were faster when preceded by a valid compared to invalid auditory cue, *t*(37) = −7.27, *p* < .001 (Figure 3a). The size of this effect was large and of similar magnitude within each age group (young adults: Cohen’s *d* = 1.21; old adults: Cohen’s *d* = 1.22). There was no significant difference in RTs between the validly and invalidly cued targets appearing after a long foreperiod, *t*(37) = .74, *p* = .47. Analysis of the cueing index did not reveal a main effect of age, *F*(1, 36) = 1.64, *p* = .21, or an interaction between foreperiod and age, *F*(1, 36) = .004, *p* = .88.[Fig-anchor fig3]

#### RSVP task

No participant performed more than 3 *SD*s beyond the mean for any condition on perceptual discrimination, so all participants were included in the analysis.

As in the blocked-design version of the task in Experiment 1, significant and equivalent effects on discriminating targets at short and long intervals were conferred by fully intermixed temporally predictive cues in both younger and older adults. Analysis of *d*′ revealed main effects of foreperiod and cue, as well as a foreperiod-by-validity interaction (*p*s ≤ .007). Age did not affect perceptual discrimination performance in this experiment (see Table 5), and there was a foreperiod-by-validity interaction in young, *F*(1, 18) = 3.71, *p* = .07, *F* test *permutation p* < .05, and in old adults, *F*(1, 19) = 4.45, *p* < .05, *F* test permutation *p* = .04. As shown in Figure 3b, identifying the target letter was easier when the cue correctly predicted its timing in the RSVP stream compared to when the cue was invalid. This was true for targets appearing after a short interval, *t*(37) = 4.22, *p* < .001 and for targets appearing after a long interval, *t*(37) = 2.55, *p* = .02, although the size of this effect was significantly larger for targets appearing at the short foreperiod (Cohen’s *d* for young adults: .68, old adults: .69) compared to long foreperiods (Cohen’s *d* for young adults: .39; old adults: .51; Figure 3b).

### Sequential Effects

#### Speeded RT task

Analysis of sequential effects was limited to trials with a valid auditory cue preceded by trials with a valid auditory cue in the trial-by-trial speeded RT task.

Analysis of the RTs revealed robust and equivalent sequential effects for detecting targets at the short interval for both younger and older adults. The ANOVA yielded a main effect of previous foreperiod and an interaction between current and previous foreperiod (*p*s < .001; see Table 6 for the key results of the ANOVA). Post hoc paired-sample *t* tests were used to inform the interaction between current foreperiod and previous foreperiod, which was significant in each age group (young adults: *F*(1, 19) = 46.06, *p* < .001, *F* test permutation *p* < .001; old adults: *F*(1, 19) = 56.90, *p* < .001, *F* test permutation *p* < .001). Responses were faster when a short foreperiod was preceded by a short compared to a long foreperiod. The effect was very large in both young (Cohen’s *d* = 1.72) and old (Cohen’s *d* = 1.43) adults. RTs were unaffected in trials with a long foreperiod, *t*(37) = −1.04, *p* = .30.[Table-anchor tbl6]

#### RSVP task

**Figure 4 fig4:**
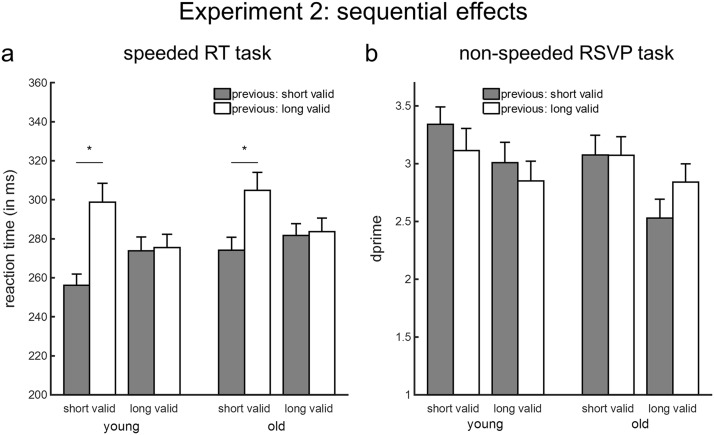
Sequential effects for the speeded reaction time (RT) task and rapid serial visual presentation (RSVP) task. Error bars represent SEM. The analysis was limited to validly cued targets preceded by validly cued targets. (a) Reaction time values (ms) in the speeded RT task. (b) Sensitivity scores (*d*′) in the RSVP task. Asterisk values denote significant effects.

Analysis of *d*′ values revealed a main effect of current foreperiod (*p* < .008), with better perceptual discrimination performance for short compared to long foreperiods, and an interaction between previous foreperiod and age (*p* = .008; see Table 6). Separate ANOVAs were run for trials with a preceding short versus long foreperiod. No main effect of age was observed when the preceding foreperiod was long, *F*(1, 36) = .01, *p* = .91. In contrast, when the previous foreperiod was short, younger adults tended to perform better than older adults, *F*(1, 36) = 3.16, *p* = .08 (Figure 4b).[Fig-anchor fig4]

In Experiment 2, we replicated the findings observed in Experiment 1 and showed that temporal expectations conferred by temporal cues can be preserved in older adults, even if older adults have to adjust their expectation from trial to trial. Although the sizes of the cued temporal expectations were equivalent in younger and older adults in both tasks across the two experiments, it is not possible to conclude that both groups of participants are affected by temporal cues in the same way. The use of only valid and invalid temporally predictive cues precludes the separation of validity benefits from invalidity costs. It is possible, for example, that younger participants proactively use cues to anticipate targets and optimize performance, whereas older adults remain more reactive and show further deficits in reorienting attention when cues are invalid. To compare the pattern of cued temporal orienting effects in the two age groups, we conducted an additional experiment. In Experiment 3, we extend the blocked-design version of the tasks to include noninformative, neutral audio cues. By comparing performance after valid and invalid temporally predictive cues relative to these neutral cues, it becomes possible to titrate the contribution of validity benefits and invalidity costs, respectively, to the overall effect of temporal orienting.

## Experiment 3: Benefits and Costs of Temporal Cues

### Method

Eighteen young (*M*_age_ 22.3, *M*_education_ = 17.8 years, 12 females) and 20 older (*M*_age_ = 68.0, *M*_education_ = 15.6 years, 12 females) volunteers took part. One older subject had to be excluded because of a score on the MoCA that indicated a mild cognitive impairment, and two additional older participants had to be excluded from the speeded RT task due to technical difficulties during data acquisition. In this experiment, we replicated the design of Experiment 1, but we added to each task two neutral blocks consisting of 48 trials each. In these blocks, targets appeared with equal probability after a short or long foreperiod, and a middle-pitched audio cue was used, which did not convey any information about the length of the upcoming foreperiod. Based on the effect sizes in younger adults observed in Experiment 1, an a priori power analysis suggested that we needed at least 5 participants in the speeded RT task and 10 participants in the RSVP task to achieve 80% power at two-sided 5% significance level to observe a significant difference between performance after valid and invalid cues (Gpower: Faul & Erdfelder, 1992).

### Results and Discussion

#### Speeded RT task

After removing the anticipatory responses, performance was at ceiling (<1% misses) for all four conditions in both age groups. One older participant was excluded from the RT analysis.

**Figure 5 fig5:**
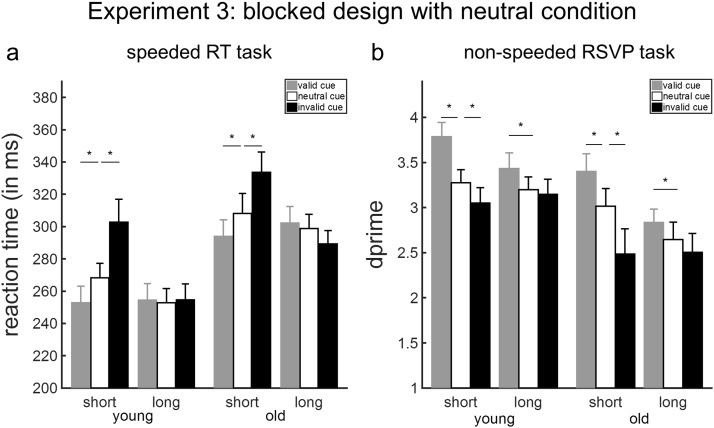
Temporal orienting effects in Experiment 3. (a) Effects of temporal expectations on reaction time (RT) values (ms) in the speeded RT task. (b) Effects of temporal expectations on sensitivity scores (*d*′) to the target items in the rapid serial visual presentation (RSVP) task. Error bars represent SEM. Asterisk values denote significant effects.

The findings indicated that blocked temporally predictive cues conferred both benefits and costs for detecting targets at the short interval in both age groups. We observed main effects of age, foreperiod, and cue validity; as well as a foreperiod-by-validity interaction on the RTs (*p*s ≤ .006; see Table 4). Post hoc paired-sample *t* tests were conducted to inform the foreperiod-by-validity interaction, which was significant within each age group (young adults: *F*(1.16, 19.81) = 15.66, G-G adj. *p* = .001, *F* test permutation *p* < .001; old adults: *F*(1.39, 20.85) = 22.58, G-G adj. *p* < .001, *F* test permutation *p* < .001). Participants reacted significantly more quickly to targets appearing after a short foreperiod when the preceding cue contained valid versus neutral temporal information, *t*(33) = −3.89, *p* < .001. The benefit of having valid temporal information was associated with a medium effect size, both in young participants (Cohen’s *d* = .61) and in old participants (Cohen’s *d* = .74; Figure 5a). In addition, participant responses were slowed down by the audio cue when it contained invalid versus neutral temporal information, *t*(33) = 5.98, *p* < .001. The cost of having invalid temporal information was associated with a large effect size, both in young participants (Cohen’s *d* = .97) and in old participants (Cohen’s *d* = 1.24; Figure 5a). The validity of the auditory cue did not modulate RTs when the foreperiod was long (valid vs. neutral: *t*(33) = .68, *p* = .50; neutral vs. invalid: *t*(33) = −1.01, *p* = .32).[Fig-anchor fig5]

Analysis of the ‘cueing index’ for validity benefits and invalidity costs did not reveal a main effect of age—benefits: *F*(1, 32) = .21, *p* = .65; costs: *F*(1, 32) = 2.53, *p* = .12—or an interaction between foreperiod and age—benefits: *F*(1, 32) = .21, *p* = .65; costs: *F*(1, 32) = .00, *p* = .98.

#### RSVP task

No participant performed more than 3 *SD*s beyond the mean for any condition on perceptual discrimination, so all participants were included in the analysis. The results showed an equivalent pattern of costs and benefits for temporally predictive cues.

A three-way ANOVA on *d*′ values showed main effects of age, foreperiod, and validity; and a foreperiod-by-validity interaction (*p*s ≤ .01, see Table 5). There were no interactions involving age (*p*s ≥ .57, see Table 5). Post hoc *t* tests were run to inform the foreperiod-by-validity interaction. When the foreperiod was short, perceptual discrimination of a target was better when the audio cue was valid relative to when it was neutral, *t*(36) = 3.37, *p* = .002 (Cohen’s *d* for young adults: .68, for old adults: .40), and worse when it was invalid compared to when it was neutral, *t*(36) = −2.21, *p* = .03 (Cohen’s *d* for young adults: .35, for old adults: .40).

For long foreperiods, we also observed a performance benefit for valid compared to neutral audio cues, *t*(36) = 2.42, *p* = .02 (Cohen’s *d* for young adults: .45, for old adults: .34). No significant difference was observed in perceptual discrimination performance between invalid versus neutral cues, *t*(36) = −.72, *p* = .48.

In summary, the introduction of blocks with noninformative neutral cues revealed that the pattern of benefits and costs conferred by blocked temporally predictive cues was equivalent for both types of task in younger and older participants. The findings suggest that older individuals are able to use temporally predictive cues proactively to anticipate targets and enhance their performance in both tasks that emphasize motor preparation and perceptual discrimination. The temporal orienting effects do not seem, therefore, to be restricted to deficits linked to excessive inflexibility or inability to reorient attention in time.

## Summary and General Discussion

We investigated the effectiveness of predictive cues in driving temporal expectations in older adults. We found no evidence that temporal orienting of attention was compromised in healthy older adults. Rather, we found robust evidence that healthy older adults generate and use temporal expectations to improve performance both in tasks that emphasize motor preparation and that emphasize perceptual discrimination. Analysis of the blocked and trial-by-trial cue designs confirmed that young and older participants benefited similarly from temporal expectations conferred by auditory cues to guide speeded responses (RT task) and perceptual discrimination (RSVP task). Inclusion of blocks with noninformative, neutral cues in the third and final experiment further demonstrated that the effects of temporal orienting in older adults come about through a similar pattern of validity benefits and invalidity costs as in the younger group. Although the benefits of temporal orienting are now very well established (for a review, see Nobre & Rohenkohl, 2014), our data represent the first demonstration of its robust preservation in aging. Our findings, therefore, question the generality of the age-related deficits reported by Zanto et al. (2011) in tasks using visual temporal orienting cues. Indirectly, our findings also constrain interpretations of age-related deficits in tasks that manipulate general temporal preparation for imperative stimuli by introducing or manipulating foreperiods (Bherer & Belleville, 2004; Gottsdanker, 1982; Vallesi, McIntosh, & Stuss, 2009; Wilkinson & Allison, 1989), suggesting that these should not be equated with temporal orienting deficits. Taken together, the findings support the growing notion that there may be several sources of temporal information acting upon stimulus processing through noncoextensive mechanisms to influence performance (see Nobre & Rohenkohl, 2014).

In our first, blocked-design experiment, the performance of older adults was on the whole less accurate and slower than that of the younger cohort, replicating previous findings (Drag & Bieliauskas, 2010; Kok, 2000; Salthouse, 2000; Vallesi al., 2009; Woodrow, 1914). Nevertheless, we observed strong cueing effects in both young and old participants in the speeded RT task, as well as in the perceptual discrimination task. Our results are consistent with previous temporal orienting studies that cue to only two moments in time (Correa, Lupiáñez, & Tudela, 2006; Coull, Frith, Büchel, & Nobre, 2000; Griffin et al., 2001), wherein the cue benefit is found to be larger in the short-foreperiod trials than in the longer-foreperiod trials. This is attributed to the predictive power of the unidirectional flow of time itself (Coull & Nobre, 1998; Nobre, 2001; Nobre, Correa, & Coull, 2007). In other words, as time passes, if the target does not occur at the short interval then participants know that it must occur at the longer one, allowing them to reorient their attention accordingly. Importantly, in our study we replicate this asymmetric cueing benefit in our speeded RT task and demonstrate that this finding is of a similar magnitude in both age groups.

In the speeded RT task, there is also evidence of the use of cues in the supplementary analyses of anticipatory responses, with participants making more anticipatory responses when they are anticipating the target to occur after a short foreperiod. Both age groups made the majority of their anticipatory responses in the invalid-cueing condition in which they expected the target to appear after a short interval, but target presentation was delayed to the long interval. These results provide converging evidence that older adults are capable of internalizing temporal expectations to aid performance.

Puzzled by the contrast between the robust temporal orienting effects in older adults in our first study, and the absence of these effects across Zanto et al.’s (2011) three task conditions, we considered whether deficits in updating information about the cue prediction on a trial-by-trial basis could have masked their temporal expectation effects. We therefore ran a trial-by-trial version of our blocked experiments to test the hypothesis that older individuals might experience a deficit in dynamically and repeatedly encoding the cue information in order to orient their attention to the relevant time point, rather than a deficit in using temporal information to improve performance per se. Support for the claim that the executive demands imposed by fully intermixing temporally predictive cues can contribute to an expectation deficit in older adults would have occurred if older participants demonstrated a validity effect in the blocked design, but not in the trial-by-trial design. The results of our second experiment, however, convincingly replicated the primary findings from our first experiment. Again, we found reliable cue validity effects for both the speeded RT task and the perceptual discrimination task.

In addition to the replication of our blocked-design results, the trial-by-trial experiment allowed us to examine sequential foreperiod effects, which also tap into the consequences of temporal structure in the environment on performance (Los, 2010). Following previous studies (Capizzi et al., 2013; Drazin, 1961; Los & Van Den Heuvel, 2001; Steinborn et al., 2008; Vallesi & Shallice, 2007), our results in the speeded RT task indicated that response times on valid trials were improved when the current foreperiod was identical to the foreperiod used in the previous trial, but only when the foreperiod was short. This asymmetrical sequential effect was present in both age groups. That is, participant RTs to early targets were faster if the previous foreperiod was short as compared to when it was long; whereas, there were similar RTs for late targets independent of whether the previous interval was short or long. The strength of the foreperiod effects in our study is all the more surprising when one considers that the effects survived the delay between trials, which included self-initiation of each trial.

Recently, it has been argued that different types of temporal expectation can be distinguished—for example, separating the effects of the sequential effects carried over across trials from other temporal orienting effects (Correa et al., 2004; Los & Van Den Heuvel, 2001). One possible interpretation is that these effects may arise from nonoverlapping mechanisms (Capizzi, Sanabria, & Correa, 2012; Triviño et al., 2010; Triviño, Arnedo, Lupiáñez, Chirivella, & Correa, 2011), whereby the observed sequential effects may be dependent on more basic and automatic mechanisms (see Vallesi et al., 2009). If this is true, then the older participants here should be thought of as having multiple types of temporal expectations intact.

The abovementioned results in the blocked and trial-by-trial studies do not support sweeping deficits in top-down control or expectations in older adults. Instead, both younger and older adults demonstrated an ability to orient their attention in time. Though older participants may be slightly slower and slightly less accurate in some contexts, our results clearly suggest that temporal expectations can be spared. This left us to question why our findings differed from those previously reported. One important difference was our use of valid versus invalid cues as compared to Zanto et al.’s (2011) use of valid versus neutral cues. The different cue-validity conditions used in our first two experiments left open the possibility that our sample of older participants did not benefit from temporal expectations conferred by the cues, but instead were hindered by breaches of expectation.

Our third experiment explored whether our findings reflected primarily invalidity costs, rather than cueing benefits. The results of our third experiment, which included neutral cues, replicated the findings in our first two experiments, confirming that older adults are able to make use of temporal cues to optimize behavioral performance — as evidenced by enhanced motor and perceptual abilities. Support for the claim that the behavioral benefits we observed in our first experiment were solely down to the costs of invalid cues would have been garnered had we found no significant difference between valid and neutral trials. Instead, our results confirmed the presence of reliable cue-validity benefits. Invalidity costs were also observed, with a similar pattern across both age groups. Our results, therefore, provide strong evidence for comparable orienting of attention in time between age groups.

Whereas researchers have argued that older participants may have deficits in inhibitory control (Gazzaley, Cooney, Rissman, & D’Esposito, 2005; Hasher, Zacks, & Rahhal, 1999), the participants in our three studies were no worse than young participants in making anticipatory responses. In fact, older adults were marginally better at inhibiting responses, with younger adults making more anticipatory responses in the late invalid conditions (see Supplementary Materials). In contrast to Pincham, Killikelly, Vuillier, and Power (2012), who argue that Zanto et al.’s (2011) results might be evidence of a deficit in proactive control, rather than expectation, the older adults in our experiments seem to be less impulsive in our two blocked design speeded RT tasks than their younger counterparts. Compared to the younger adults, the older adults in our study are more inhibited and less reactive.

In line with the age differences observed in the RT effects on our blocked design experiments, these results are at least somewhat consistent with the research that reports an age-related slowing on speeded RT tasks (Salthouse, 1996), and are consistent with the suggestion that older adults tend to be more cautious (Lindenberger & Mayr, 2014). However, the precise explanation for these age-related differences in the number of anticipatory responses remains difficult to pinpoint, and could reflect a mixture of age-related differences: in overall impulsivity and inhibition (Connelly & Hasher, 1993; Hasher, Stoltzfus, Zacks, & Rypma, 1991; Tipper, 1991); the extent to which the two age groups internalize predictions by the cues (e.g., reflecting differences in top-down control, see Zanto and Gazzaley (2014) for review); the rate or efficiency with which the groups can orient or reorient attention (see Cona, Bisiacchi, Amodio, & Schiff, 2013); and/or the faster speed of young participants’ responses constraining the ability to withhold a prepotent response.

It is important to consider the possible influence of our task parameters in unveiling cued temporal expectation effects in the older participants. Traditionally, the aging literature focuses almost exclusively on unimodal paradigms (e.g., visual or auditory) when comparing young and old performance (for review see Guerreiro, Murphy, & Van Gerven, 2010). In contrast, our task design used auditory cues to guide visual temporal orienting. It may be that auditory cues are more intuitive or more efficient at guiding temporal predictions. Alternatively, visual cues may cause interference with the visual target, whereas auditory cues do not engage the visual system and are therefore less disruptive. Some support for this can be seen from data showing that older adults take longer to disengage from visual stimuli compared to younger adults (e.g., Cona et al., 2013) and age-related performance declines due to distraction are minimal (if at all) when presented with relevant/irrelevant stimuli in different modalities (i.e., auditory and visual; reviewed in Guerreiro et al., 2010). By increasing task demands, or experimenting with different types of cues, future studies could look to separate these effects and explore the extent to which the salience and task appropriateness of the cue might contribute to differences in behavioral outcomes. To the extent that extraneous task variables have deleterious effects, it would be prudent to revisit other types of expectation deficits in the elderly.

Finally, task performance in different age groups may vary as a consequence of different strategies or state variables, such as attentional time-sharing (e.g., decreased divided attention), fatigue, or boredom (Lustig, 2003; Lustig & Meck, 2001). It is possible, although somewhat speculative, that our participants were less fatigued than those in Zanto et al.’s (2011) study, which involved a lengthier set-up time for EEG investigation. Demographic variables, such as years of education, have also been suggested to influence the preservation of certain cognitive abilities—though this is in itself debatable (Anstey & Christensen, 2000; Zahodne et al., 2011). On the surface, our older cohort had similar levels of education and performance levels on neuropsychological tests as those in Zanto et al.’s study (2011), though it remains possible that subtle differences in some other demographic or neuropsychological factor might contribute to the different nature of our findings.

Being able to use top-down control proactively to stay focused and optimize performance in the task at hand is essential to cognitive well-being. Building on our promising findings, it will be important to continue to explore the boundaries of conditions that enable older adults to make use of temporal cues. Moreover, given that we have identified a task in which both older and younger adults are able to use temporal information to orient their attention and suppress irrelevant or distracting information (i.e., in the case of the perceptual discrimination task), future studies could use our paradigm to explore the neural mechanisms involved in temporal expectation, the degree to which they are separable, and their preservation in normal aging.

## Conclusion

If age-related differences in temporal expectation exist, it is probably too early to conclude that older adults suffer from a categorical deficit. The deviation of our findings from Zanto et al.’s (2011) suggests that more work ought to be done to uncover the conditions for an expectation deficiency. That is, to the extent that research into temporal expectation in aging is motivated by designing aging-friendly environments, aging research must involve an investigation into the boundaries of aging-related shifts in internal-to-external processing. For instance, the use of simple cues that provide intuitive stimulus driven associations may be more appropriate as an aid to guide behavior in various adaptive contexts. Our results provide the first demonstration that temporal expectation can work on its own to enhance behavioral performance well into old age. More work ought to be done to ascertain the point at which temporal orienting of attention declines in normal aging, and to uncover the limits of this effect in healthy aging.

## Supplementary Material

10.1037/pag0000105.supp

## Figures and Tables

**Table 1 tbl1:** Neuropsychological Evaluation Conducted in Older Adults From Experiments 1 and 2 (N = 26, 11F)

Variable	*M*	Range	*SE*	*N* > 2 *SD*^a^
Age	66.4	61–82	1.0	0
Education	18.0	11–29	.8	0
MoCA	28.0	26–30	.2	0
TMT: A (s)	32.1	16–68	1.9	0
TMT: B (s)	66.8	41–160	4.8	0
ROCFT				0
Copy (out of a possible 36)	34.1	30–36	.4	0
Immediate (out of a possible 36)	20.5	3.5–34	1.2	1
Delay (out of a possible 36)	18.9	2.5–34	1.3	0
Category Fluency	21.9	15–34	1.0	0
HVLT-R				
Trial 1	5.5	3–9	.3	0
Trial 2	8.1	3–12	.4	0
Trial 3	9.6	6–12	.3	0
Learning	4.2	1–7	.3	0
Sum of 1–3	23.2	13–32	.8	0
Delayed recall	8.3	0–12	.5	1
Percent retained (%)	83.6	0–110	4.5	1
True positives	11.0	7–13	.3	1
False positives	.7	0–3	.2	0
Discrimination index	10.4	7–13	.3	0
BNT	14.5	12–15	.2	0
Digit span (scaled score)	10.4	6–14	.5	n.a.
Purdue pegboard right	12.5	9–17	.4	0
Purdue pegboard left	12.4	9–17	.4	1
Purdue pegboard both	10.3	8–13	.3	0
Purdue pegboard sum	35.2	28–44	.9	3
Purdue pegboard assembly	26.0	15–33	.9	1
TOPF-FSIQ	120.3	102–138.9	2.1	0
*Note.* F = females; MoCA = Montreal Cognitive Assessment; TMT = Trail Making Test; ROCFT = Rey-Osterrieth Complex Fig. Test; HVLT-R = Hopkins Verbal Learning Test—Revised; BNT = 15-Item Boston Naming Test; TOPF-FSIQ = Test of Premorbid Functioning - Full Scale Intelligence Quotient.				
^a^ This column contains the number of individuals who had a score that was two standard deviations away from the age-adjusted normative values (n.a. = relevant normative values not available).				

**Table 2 tbl2:** Mean Scores From the Neuropsychological Evaluation Conducted for the Old and Young Adults Who Participated in Experiment 3

	Old (*n* = 19, 11F)				Young (*n* = 18, 12F)				
Variable	*M*	Range	*SE*	>2 *SD*^a^	*M*	Range	*SE*	>2 *SD*^a^	*P*
Age	67.1	51–83	2.0	0	22.7	19–28	.6	0	<.001
Education	15.6	10–24	.8	0	17.8	13–23	.6	0	<.05
MoCA	28.1	26–30	.3	0	27.6	26–30	.3	0	*n.s.*
TMT: A (s)	32.9	18–87	3.9	1	23.6	12–49	2.4	1	<.05
TMT: B (s)	78.8	32–178	9.4	2	50.8	22–183	8.5	1	<.01
ROCFT									
Copy (out of a possible 36)	31.5	19–36	.9	1	33.5	28–36	.5	n.a.	*n.s.*
Immediate (out of a possible 36)	16.2	6–27	1.5	0	20.6	12.5–28	1.3	n.a.	*n.s.*
Delay (out of a possible 36)	14.7	3–27	1.7	1	20.6	10–28.5	1.5	n.a.	<.05
Category fluency	22.9	6–24	.8	0	23.1	9–34	1.5	1	*n.s.*
HVLT-R									
Trial 1	5.7	2–9	.4	0	5.8	3–8	.3	n.a.	*n.s.*
Trial 2	8.1	4–11	.4	0	8.7	6–12	.4	n.a.	*n.s.*
Trial 3	9.3	6–11	.4	0	10.3	7–12	.4	n.a.	<.05
Learning	3.6	0–7	.5	0	4.6	2–7	.3	n.a.	*n.s.*
Sum of 1–3	23.1	13–29	.9	0	24.8	16–32	24.8	n.a.	*n.s.*
Delayed recall	8.7	4–12	.6	0	9.2	6–12	.5	n.a.	*n.s.*
Percent retained (%)	94.6	44–133	5.2	0	89.2	50–109	3.4	n.a.	*n.s.*
True positives	11.3	9–12	.2	1	11.7	10–12	.1	n.a.	*n.s.*
False positives	1.2	0–13	.3	0	.4	0–2	.1	n.a.	<.05
Discrimination index	10.2	7–12	.4	0	11.3	9–12	.2	n.a.	*n.s.*
BNT	14.2	12–15	.2	0	11.8	7–14	.4	n.a.	<.001
Digit span (scaled score)	10.7	7–16	.6	n.a.	9.3	5–17	.6	n.a.	*n.s.*
Purdue pegboard right	12.4	7–19	.6	1	14.3	12–18	.4	1	<.01
Purdue pegboard left	11.6	9–15	.4	1	13.0	10–17	.5	5	*n.s.*
Purdue pegboard both	9.5	5–12	.5	3	11.4	9–15	.4	4	<.01
Purdue pegboard assembly	24.6	14–37	1.5	3	32.1	20–48	1.8	6	<.01
TOPF-FSIQ	114.3	96.9–132.5	2.4	0	110.6	101.1–120.1	1.3	0	*n.s.*
*Note.* F = females; MoCA = Montreal Cognitive Assessment; TMT = Trail Making Test; ROCFT, Rey-Osterrieth Complex Fig. Test; HVLT-R = Hopkins Verbal Learning Test-Revised; BNT = 15-Item Boston Naming Test; TOPF-FSIQ = Test of Premorbid Functioning-Full Scale Intelligence Quotient; *P* = probability of difference between young and old adults, Mann-Whitney nonparametric test (*n.s.*, non-significant).									
^a^ These columns contain the number of individuals who had a score that was two standard deviations away from the age-adjusted normative values (n.a. = relevant normative values not available).									

**Table 3 tbl3:** The Number of Excluded Outlying Trials per Experiment

	Old adults			Young adults			Two-sample *t*-test
Experiment	*M*	Min	Max	*M*	Min	Max	
Speeded RT task							
Experiment 1	5.83	1	11	5.16	1	10	*t*(34) = .77, *p* = .44
Experiment 2	5.50	1	11	7.17	2	10	*t*(36) = −1.91, *p* = .06
Experiment 3	6.64	1	12	7.06	2	21	*t*(33) = −.33, *p* = .76
RSVP task							
Experiment 1	7.78	1	13	7.72	4	13	*t*(34) = .06, *p* = .95
Experiment 2	7.55	1	13	7.00	2	11	*t*(36) = .56, *p* = .58
Experiment 3	8.74	1	15	9.00	4	16	*t*(35) = −.98, *p* = .33
*Note*. With reaction time (RT) values more than 3 *SD*s away from the mean RT of a participant classified as outlying. The average number of outlying trials did not differ between both age groups, according to two-sample *t*-tests. RSVP = rapid serial visual presentation.							

**Table 4 tbl4:** Analysis of Variance on Reaction Time (RT) Values From the Speeded RT Task

Effect	*df*1	*df*2	*F*	*p*	η^2^
Experiment 1					
Age^a^	1	32	23.82	<.001	.42
Foreperiod^a^	1	32	55.56	<.001	.64
Foreperiod × Age	1	32	.31	.58	
Validity^a^	1	32	79.97	<.001	.71
Validity × Age	1	32	.001	.97	
Foreperiod × Validity^a^	1	32	114.25	<.001	.78
Foreperiod × Validity × Age	1	32	.33	.57	
Experiment 2					
Age	1	36	1.70	.20	
Foreperiod^a^	1	36	55.10	<.001	.64
Foreperiod × Age	1	36	2.33	.14	
Validity^a^	1	36	52.68	<.001	.60
Validity × Age	1	36	2.31	.14	
Foreperiod × Validity^a^	1	36	43.37	<.001	.55
Foreperiod × Validity × Age	1	36	1.35	.25	
Experiment 3					
Age^a^	1	32	8.62	.006	.32
Foreperiod^a^	1	32	24.16	<.001	.43
Foreperiod × Age	1	32	.57	.46	
Validity^a^	1.66	53.25	20.91	<.001^b^	.43
Validity × Age	1.66	53.25	2.18	.13^b^	
Foreperiod × Validity^a^	1.25	39.91	36.47	<.001^b^	.53
Foreperiod × Validity × Age	1.25	42.05	.03	.98^b^	
^a^ Significant effects. ^b^ Greenhouse-Geisser adjusted *p* value.					

**Table 5 tbl5:** Analysis of Variance on d′ Values From the RSVP Task

Effect	*df*1	*df*2	*F*	*p*	η^2^
Experiment 1					
Age^a^	1	34	8.44	.006	.20
Foreperiod	1	34	2.00	.17	
Foreperiod × Age	1	34	2.29	.14	
Validity^a^	1	34	43.26	<.001	.56
Validity × Age	1	34	1.19	.28	
Foreperiod × Validity	1	34	2.41	.13	
Foreperiod × Validity × Age	1	34	3.43	.07	
Experiment 2					
Age	1	36	.09	.77	
Foreperiod^a^	1	36	10.99	.002	.23
Foreperiod × Age	1	36	1.06	.31	
Validity^a^	1	36	13.38	.002	.27
Validity × Age	1	36	.35	.56	
Foreperiod × Validity^a^	1	36	8.12	.007	.18
Foreperiod × Validity × Age	1	36	.02	.90	
Experiment 3					
Age^a^	1	35	7.23	.01	.17
Foreperiod^a^	1	35	5.37	.03	.13
Foreperiod × Age	1	35	1.18	.28	
Validity^a^	1.83	63.92	13.67	<.001^b^	.28
Validity × Age	1.83	63.92	.42	.64b	
Foreperiod × Validity^a^	1.66	58.25	4.11	.03^b^	.11
Foreperiod × Validity × Age	1.66	58.25	.19	.79^b^	
*Note*. RSVP = rapid serial visual presentation.					
^a^ Significant effects. ^b^ Greenhouse-Geisser adjusted *p* value.					

**Table 6 tbl6:** Experiment 2: Analysis of Variance on the Sequential Effects

Effect	*df*1	*df*2	*F*	*p*	η^2^
Speeded RT task					
Age	1	36	1.08	.31	
Current Foreperiod	1	36	2.74	.11	
Current Foreperiod × Age	1	36	.47	.50	
Previous Foreperiod^a^	1	36	64.90	<.001	.64
Previous Foreperiod × Age	1	36	1.44	.24	
Current Foreperiod × Previous Foreperiod^a^	1	36	99.34	<.001	.73
Current Foreperiod × Previous	1	36	3.23	.09	
Foreperiod × Age					
RSVP task					
Age	1	36	.95	.34	
Current Foreperiod^a^	1	36	14.66	<.001	.29
Current Foreperiod × Age	1	36	.26	.62	
Previous Foreperiod	1	36	.09	.77	
Previous Foreperiod × Age^a^	1	36	8.02	.008	.18
Current Foreperiod × Previous Foreperiod	1	36	2.64	.11	
Current Foreperiod × Previous Foreperiod × Age	1	36	1.08	.31	
*Note*. RT = reaction time; RSVP = rapid serial visual presentation.					
^a^ Significant effects.
