# Supplementary material for: Temporal Orienting of Attention Can Be Preserved in Normal Aging
Source: Psychol Aging. 2016 Jun 13;31(5):442–55. doi: 10.1037/pag0000105 (PMC4976797; doi:10.1037/pag0000105)
Supplement: Supplementary file 1 [file z2m-2989_PAG-2015-0418_SUPPL.zip › z2m004162989sb01.docx]

**Supplemental Materials**

**Temporal Orienting of Attention can be Preserved in Normal Aging**

**by J. J. Chauvin et al., 2016, *Psychology and Aging***

**http://dx.doi.org/10.1037/pag0000105**

**Supplementary Methods**

**Supplementary Outcome Variables**

In addition to our primary outcome variables, the response times in the speeded reaction time (RT) task and the perceptual discrimination values (*d’*) in the rapid serial visual presentation (RSVP) task, we analyzed the proportion of anticipatory responses in the speeded RT task and the response times in the RSVP task. Anticipatory responses were defined as either responses occurring before the onset of the target or responses with a RT less than 100 ms. RTs in the non-speeded RSVP task were adjusted according to the participant’s accuracy using the inverse efficiency measure (Chambers, Stokes, & Mattingley, 2004; Romei, Driver, Schyns, & Thut, 2011; Townsend & Ashby, 1983). The inverse efficiency score (IES) was calculated by dividing the mean reaction time by the proportion of correct responses.

For each supplementary measure, we excluded from the analysis participants who scored more than three standard deviations away from the mean value in at least one condition. To examine how sensitivity to temporal information changed with age, we ran a 3-way mixed-design analysis-of-variance (ANOVA) with foreperiod (short, long) and cue validity (Experiments 1 and 2: valid, invalid; Experiment 3: valid, invalid, neutral) as within-subjects factor, and age group (young, old) as a between-subjects factor for each task. When sphericity could not be assumed (Mauchly’s sphericity test: *p* < .05), *p*-values were adjusted using the Greenhouse-Geisser correction (G-G correction).

**Supplementary Analysis: Blocked versus Trial-by-Trial Design**

In order to explore whether differences in performance depended on whether the auditory cues were blocked, we ran a 4-way analysis of variance with study (‘blocked design’, ‘trial-by-trial design’) as between-subjects factor. Only older participants who participated in both studies were included in the analysis (*n* = 13).

**Supplementary Results**

**Experiment 1: Temporal Orienting in a Blocked Design**

**Anticipatory responses in the speeded RT task.** Two participants (one young, one old) were excluded from the analysis on the proportion of anticipatory responses. This was because the proportion of their anticipatory responses was more than 3 standard deviations (SD) higher than the average proportion of anticipatory responses across all the other participants.

Both groups of participants committed a significantly larger number of anticipatory responses when they were cued to expect the target at the short foreperiod. We observed main effects of foreperiod (*F*(1,32) = 29.55, *p* < .001) and cue validity (*F*(1,32) = 20.15, *p* < .001), and a foreperiod-by-validity interaction (*F*(1,32) = 27.62, *p* < .001) on the proportion of anticipatory responses (Supplementary Figure 1a). Post-hoc *t-*tests were conducted to inform the foreperiod-by-validity interaction. Anticipatory responses on late targets were more frequent when participants expected a short foreperiod (invalid cue) compared to when they expected a long foreperiod (valid cue) (*t*(33) = -5.02, *p* < .001). The validity of the cue had an opposite effect on the proportion of anticipatory responses when the foreperiod was short: participants made more anticipatory responses when a short (valid cue) compared to a long foreperiod (invalid cue) was expected (*t*(33) = 4.40, *p* < .001) (Supplementary Figure 1).

In addition, there was an age-by-foreperiod (*F*(1,32) = 9.06, *p* = .005) and an age-by-validity interaction (*F*(1,32) = 6.16, *p* = .02), but no three-way interaction (*F*(1,32) = 1.38, *p* = .25). The age-by-foreperiod interaction was further examined by running separate ANOVAs for the early versus late foreperiod. When the target appeared late, younger individuals tended to make more anticipatory responses than older individuals (main effect of age: *F*(1,32) = 3.55, *p* = .07), whereas no such trend was observed for short foreperiods (main effect of age: *F*(1,32) = 1.77, *p* = .19). The age-by-validity interaction was further explored by running separate ANOVAs for valid versus invalid auditory cues. Younger individuals tended to make more anticipatory responses than older individuals when the cues were invalid (*F*(1,32) = 2.61, *p =* .12), but no such trend was present for valid cues (*F*(1,32) = .07, *p* = .80) (Supplementary Figure 1a).

**
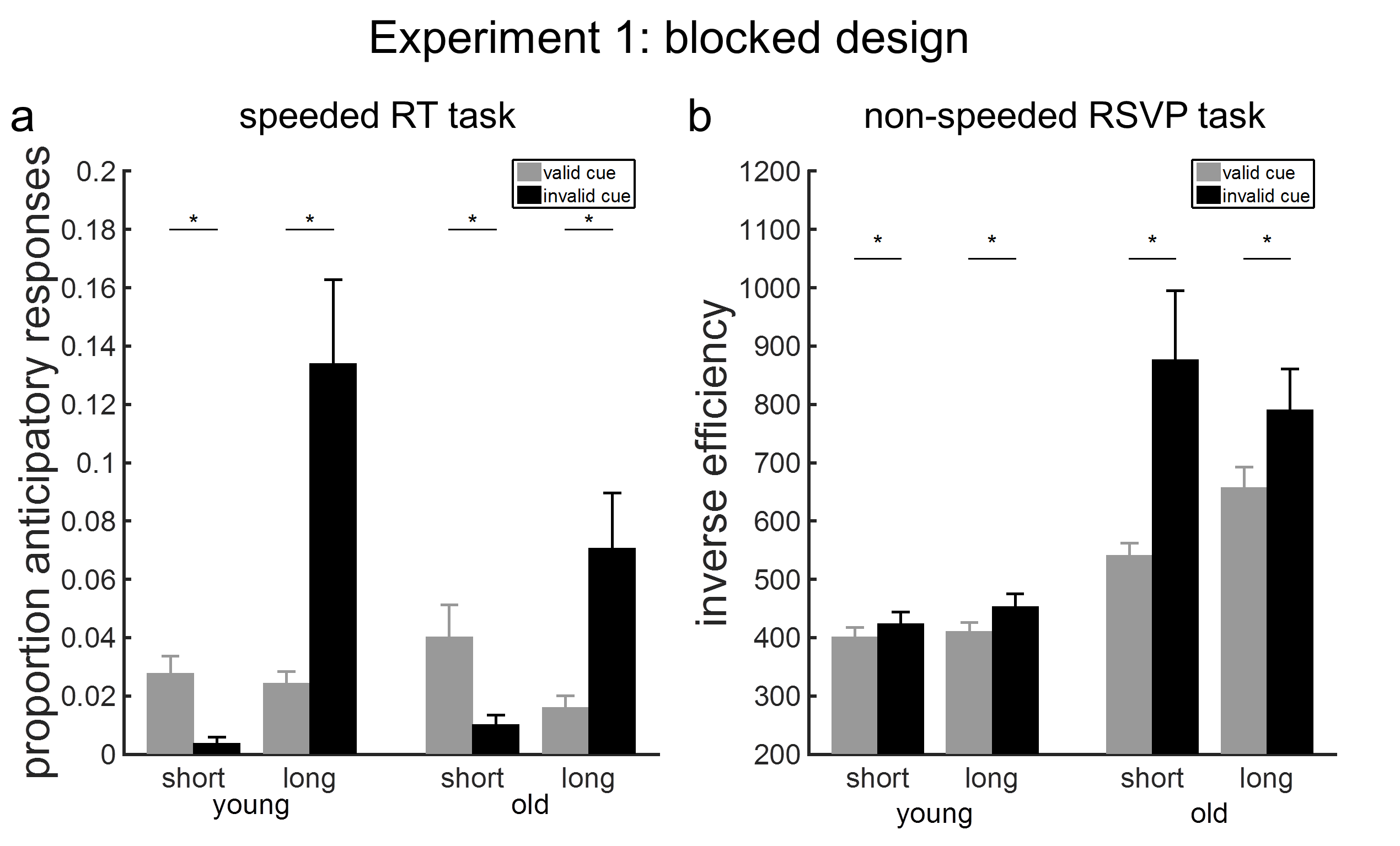
***Supplementary Figure 1.* Temporal orienting effects in Experiment 1 (blocked design). (a) Effects of temporal expectations on the proportion of anticipatory responses in the speeded RT task. (b) Effects of temporal expectations on sensitivity scores (*d’*) to the target items in the RSVP task. Error bars represent SEM.

**Response times in the RSVP task.** One older participant was excluded from the analysis because of high RTs.

Since the omnibus ANOVA revealed a three-way interaction between foreperiod, validity, and age (*F*(1,33) = 8.28, *p* = .04), we ran separate ANOVAs for short versus long foreperiods. When the target appeared early, the main effects of age (*F*(1,33) = 19.94, *p* < .001) and validity (*F*(1,33) = 10.76, *p* = .002), as well as the two-way interaction (*F*(1,33) = 8.26, *p* = .007) were significant. Post-hoc independent sample *t-*tests revealed that older individuals responded more slowly compared to younger adults independent of the cue type, but the magnitude of the effect was larger for invalid compared to valid cues (invalid cue: *t*(33) = 4.97, *p* < .001, Cohen’s *d* = 1.67*;* valid cue: *t*(33) = 3.82, *p* = .001, Cohen’s *d* = 1.26*)* (Supplementary Figure 1b). When the foreperiod was long, we observed main effects of validity (*F*(1,33) =8.27, *p* = .007) and age (*F*(1,33) = 35.77, *p* < .001), but no significant age-by-validity interaction (*F*(1,33) = 2.11, *p* = .16).


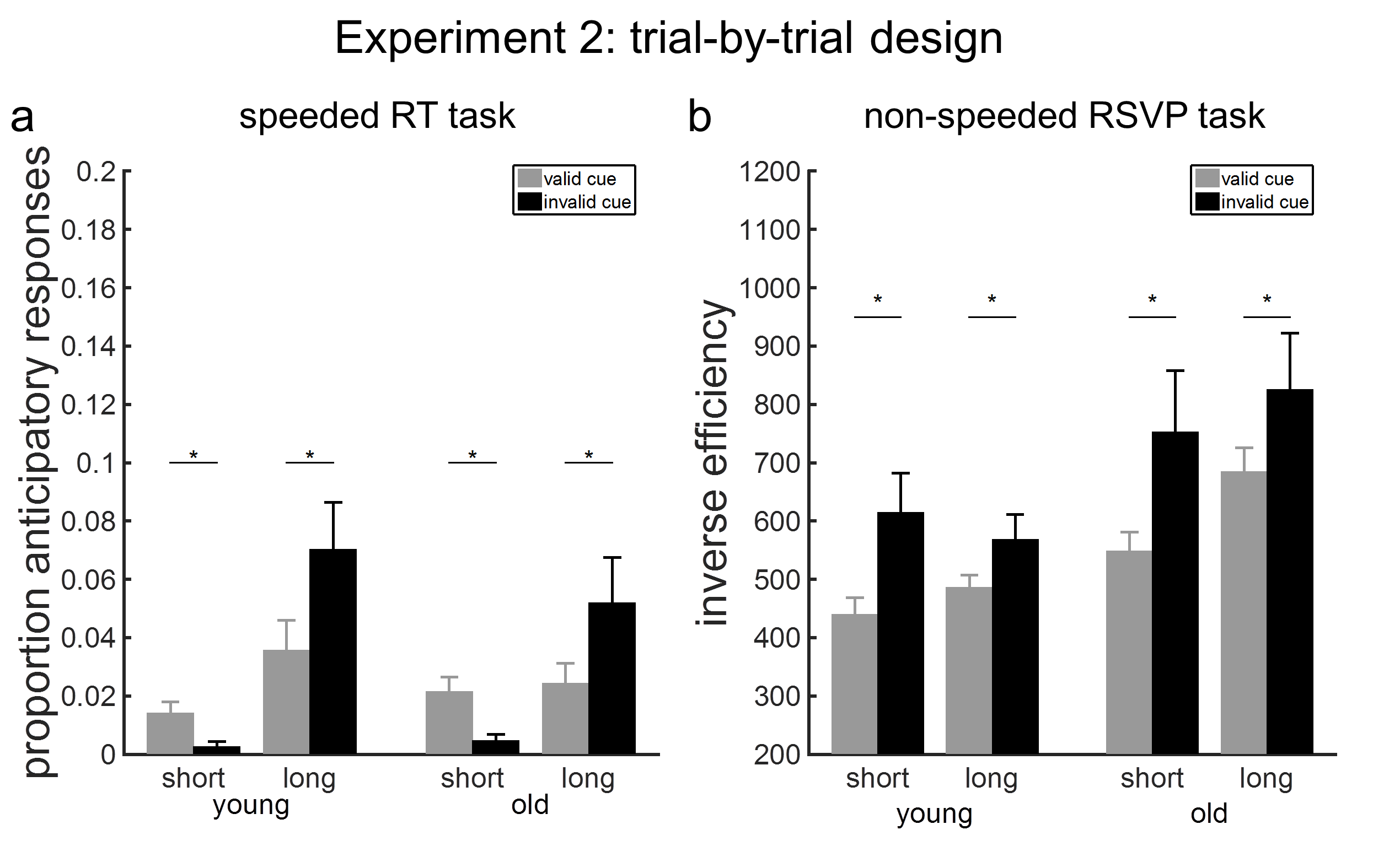
*Supplementary Figure 2.* Temporal orienting effects in Experiment 2 (trial-by-trial design). (a) Effects of temporal expectations on the proportion of anticipatory responses in the speeded RT task. (b) Effects of temporal expectations on sensitivity scores (*d’*) to the target items in the RSVP task. Error bars represent SEM.

**Experiment 2: Temporal Orienting in a Trial-by-Trial Design**

**Anticipatory responses in the speeded RT task.** One older participant was excluded because of a high proportion of anticipatory responses.

Younger and older participants showed a similar pattern of anticipatory responses when predictive cues were fully intermixed. As in Experiment 1, anticipatory responses were accentuated when cues predicted the target to appear after the short foreperiod. The three-way ANOVA revealed a main effect of foreperiod (*F*(1,34) = 28.29, *p* < .001) and a foreperiod-by-validity interaction (*F*(1,34) = 13.96, *p* = .001). No other effect was significant (main effect of age: *F*(1,34) = .34, *p* = .57; age-by-foreperiod interaction: *F*(1,34) = 2.24, *p* = .14; main effect of validity: *F*(1,34) = 3.62, *p* = .07; age-by-validity interaction: *F*(1,34) = .49, *p* = .49; age-by-foreperiod-by-validity interaction: *F*(1,34) = .004, *p* = .95). Post-hoc paired-sample *t*-tests showed that when the target appeared late, the proportion of anticipatory responses was higher when the cue was invalid rather than valid (*t*(35) = -3.14, *p* = .003). In contrast, when the target appeared early, the proportion of anticipatory responses was higher when the cue contained valid, instead of invalid temporal information (*t*(35) = 4.03, *p* < .001). (Supplementary Figure 2a).

**Response times in the RSVP task.**  Two participants (one old, one young) were excluded because of high response times. All participants were included in the analysis. Analysis of RTs using the inverse efficiency measure showed main effects of foreperiod (*F*(1,34) = 17.34, *p* < .001) and cue validity (*F*(1,34) = 7.59, *p* = .009), as well as a foreperiod-by-validity interaction (*F*(1,34) =12.83, *p* = .001). We also observed a main effect of age (*F*(1,34) = 6.32, *p* = .02) and an age-by-foreperiod interaction (*F*(1,34) = 17.41, *p* < .001), but no age-by-validity interaction (*F*(1,34) = .16, *p* = .69) and no age-by-foreperiod-by-validity interaction (*F*(1,34) = .42, *p* = .52). RTs were shorter when the auditory cue contained valid compared to invalid temporal information. The foreperiod-by-validity interaction indicated that the magnitude of this effect was larger for short (*t*(35) = -3.11, *p* = .004) compared to long foreperiods (*t*(35) = -2.26, *p* = .02) (Supplementary Figure 2b).

To test the age-by-foreperiod interaction, we ran separate ANOVAs for short and long foreperiods. When the target appeared after a long interval, older participants responded more slowly compared to young adults (*F*(1,34) = 10.90, *p* = 0.002), but age did not modulate response times when the target appeared after a short interval, or did so only marginally (*F*(1,34) = 2.87, *p* = .10).

**Experiment 3: Benefits and Costs of Temporal Cues**

**Anticipatory responses in the speeded RT task.** Three participants (two young, one old) were excluded from the analysis on the proportion of anticipatory responses.

We observed a three-way interaction between age, validity and foreperiod (*F*(1.38, 41.30) = 7.06, G-G adj. *p* = .006). To understand this interaction, we ran separate two-way ANOVAs for short and long foreperiods. For short foreperiods, we observed a significant main effect of validity (*F*(1.62, 48.62) = 19.89, G-G adj. *p* < .001), but no main effect of age (*F*(1,30) = .00, *p* = .99) and no age-by-validity interaction (*F*(1.62,48.62) = 3.04, G-G adj. *p* = 0.07). Post-hoc *t*-tests showed that anticipatory responses were more frequent for valid versus neutral audio cues (*t*(31) = 4.80, *p* < .001), whereas there was no significant difference in anticipatory responses for invalid versus neutral cues (*t*(31) = -0.76, *p* = 0.45).


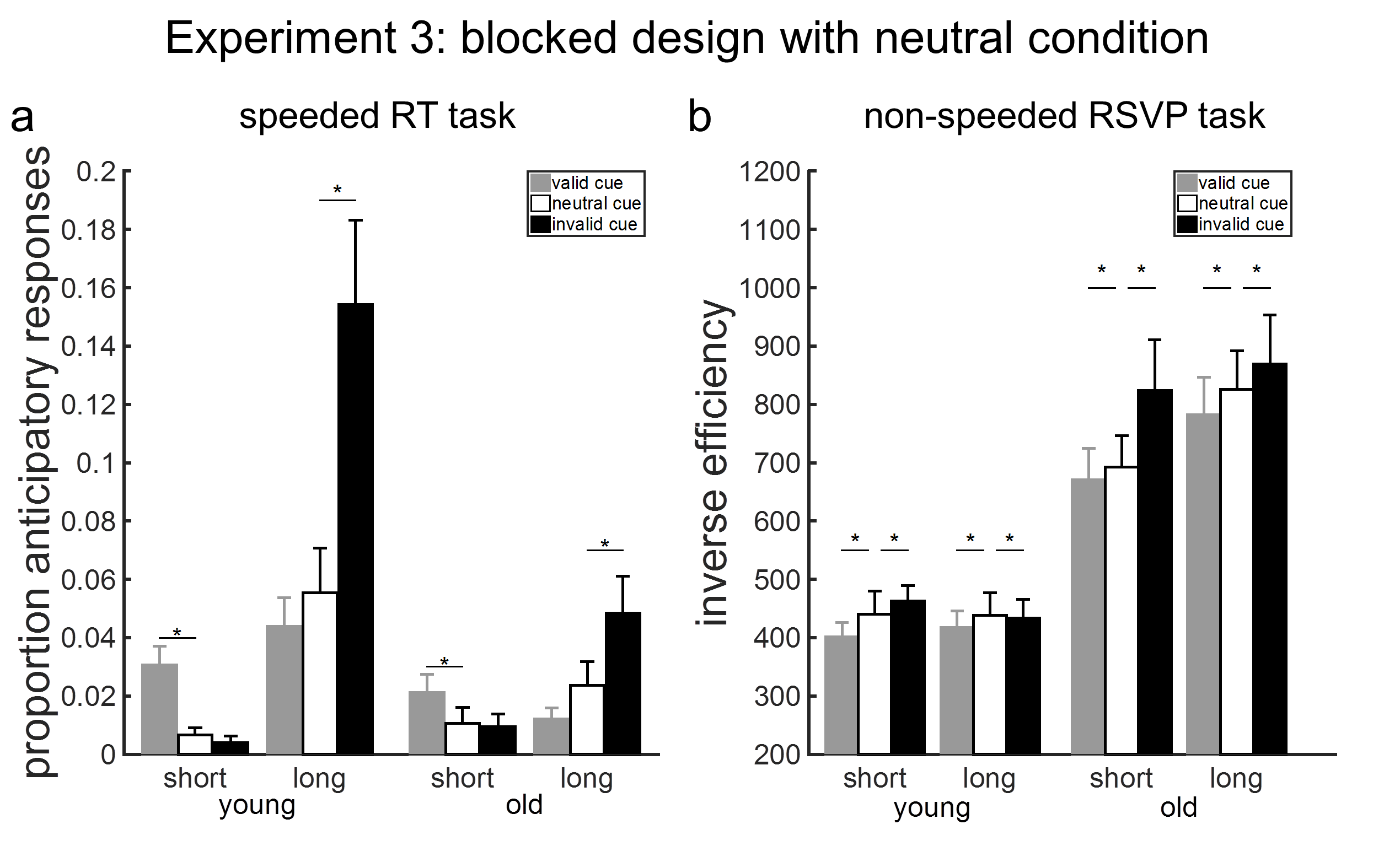


*Supplementary Figure 3.* Temporal orienting effects in Experiment 3. (a) Effects of temporal expectations on the proportion of anticipatory responses in the speeded RT task. (b) Effects of temporal expectations on sensitivity scores (*d’*) to the target items in the RSVP task. Error bars represent SEM.

A two-way ANOVA limited to the long foreperiod showed main effects of age (*F*(1,30) = 10.66, *p* = .003) and validity (*F*(1.55, 46.45) = 24.30, *p* < .001) as well as an age-by-validity interaction (*F*(1.55, 46.45) = 7.15, *p* = .004). Post-hoc independent sample *t-*tests demonstrated that younger adults had a higher proportion of anticipatory responses when the cue was valid (*t*(30) = 2.98, *p* = .006) or invalid (*t*(30) = 3.36, *p* =.002), but not when the cue was neutral (*t*(30) = 1.83, *p* = .08).

**Response times in the RSVP task.** Two old participants were excluded because of high response times. We observed main effects of age (*F*(1,33) = 25.63, *p* < .001), foreperiod (*F*(1,33) = 10.81, *p* = .002) and validity (*F*(1.56,51.38) = 8.81, G-G adj. *p* = .001). We also observed an age-by-foreperiod interaction (*F*(1,33) = 13.40, *p* = .001), but no age-by-validity interaction (*F*(1.56,51.38) = 2.96, G-G adj. *p* = .08), no foreperiod-by-validity interaction (*F*(1.37,45.24) = 2.38, G-G adj. *p* = .12), and no three-way interaction between age, foreperiod, and validity (*F*(1.37,45.24 = .53, G-G adj. *p =* .53).

Post-hoc *t-*tests to separate the patterns of validity benefits and invalidity costs showed that participants were faster when the audio cue preceding the target was valid compared to neutral (*t*(34) = -2.43, *p* = .02), but slower when the cue was invalid relative to neutral (*t*(34) = 3.13, *p* = .04). The interaction between age and foreperiod was further investigated by running separate ANOVAs for each foreperiod. Younger participants were significantly faster than older participants for both foreperiods, but the effect was larger for the long compared to the short foreperiod (short foreperiod: *F*(1,33) = 20.22, *p* < .001; long foreperiod: *F*(1,33) = 29.03, *p* < .001).

**Blocked versus Trial-by-Trial Design**

**Speeded RT task.** This analysis was limited to the thirteen older participants who took part in Experiments 1 and 2.

A within-subjects ANOVA with design (‘blocked’, ‘trial-by-trial’), foreperiod, and cue validity as factors and the mean RT as outcome variable (Supplementary Figure 1a versus Supplementary Figure 2a) revealed a significant main effect of design (*F*(1,12) = 7.59, *p* = .02), as well as a significant three-way interaction (*F*(1,12) = 14.95, *p* = .002) (see Supplementary Table 1). This result suggests that RTs were longer, and the asymmetric cueing benefit stronger, in the blocked compared to the trial-by-trial version of the task. This is in line with the additional sources of temporal predictions in the blocked vs. trial-by-trial design. Only in the blocked design is there additional information related to tonic differences in the conditional probability for targets to appear at short versus long foreperiods.

**RSVP task.**  Perceptual sensitivity in the RSVP task was not dependent on whether the temporal cues were blocked, nor did the design interact with any other factor (Supplementary Table 1).

Supplementary Table 1

*Analysis of variance (ANOVA) to compare the blocked design (Experiment 1) to the trial-by-trial design (Experiment 2)*

| Effect | df1 | df2 | *F* | *p* | *η^2^* |
| --- | --- | --- | --- | --- | --- |
| **RTs in the Speeded RT task** |  |  |  |  |  |
| Experimental design* | 1 | 12 | 7.59 | .02 | .39 |
| Foreperiod* | 1 | 12 | 35.06 | <.001 | .75 |
| Validity* | 1 | 12 | 37.23 | <.001 | .76 |
| Experimental design x Foreperiod | 1 | 12 | 2.48 | .14 |  |
| Experimental design x Validity* | 1 | 12 | 5.77 | .03 | .33 |
| Foreperiod x Validity* | 1 | 12 | 104.73 | <.001 | .90 |
| Experimental design x Foreperiod  x Age* | 1 | 12 | 14.95 | .002 | .56 |
| ***d’* in the RSVP task** |  |  |  |  |  |
| Experimental design | 1 | 12 | .69 | .42 |  |
| Foreperiod* | 1 | 12 | 8.20 | .01 | .41 |
| Validity* | 1 | 12 | 19.82 | .001 | .62 |
| Experimental design x Foreperiod | 1 | 12 | .09 | .77 |  |
| Experimental design x Validity | 1 | 12 | .28 | .61 |  |
| Foreperiod x Validity* | 1 | 12 | 6.10 | .03 | .34 |
| Experimental design x Foreperiod  x Age | 1 | 12 | .48 | .50 |  |

*Note.* * = significant effects.

**Reference List**

Chambers, C. D., Stokes, M. G., & Mattingley J. B. (2004). Modality-specific control of strategic spatial attention in parietal cortex. *Neuron*, 44 (6), 925–930.

Romei, V., Driver, J., Schyns, P. G., & Thut, G. (2011). Rhythmic TMS over parietal cortex links distinct brain frequencies to global versus local visual processing. *Current Biology*, 21 (4), 334–337.

Townsend, J. T. & Ashby, F. G. (1983). *Stochastic modelling of elementary psychological processes*. London: Cambridge University Press.
